# KHDRBS1 as a novel prognostic signaling biomarker influencing hepatocellular carcinoma cell proliferation, migration, immune microenvironment, and drug sensitivity

**DOI:** 10.3389/fimmu.2024.1393801

**Published:** 2024-04-10

**Authors:** Rui Fan, Fahui Liu, Qiming Gong, Donghua Liu, Shihang Tang, Dongyan Shen

**Affiliations:** ^1^ Xiamen Cell Therapy Research Center, The First Affiliated Hospital of Xiamen University, School of Medicine, Xiamen University, Xiamen, China; ^2^ Department of Nephrology, Affiliated Hospital of Youjiang Medical University for Nationalities, Baise, China; ^3^ Baise Key Laboratory for Metabolic Diseases (Youjiang Medical University for Nationalities), Education Department of Guangxi Zhuang Autonomous Region, Baise, China

**Keywords:** HCC, KHDRBS1, immune, migration, multi-omics, biomarker

## Abstract

**Background:**

Human tumors pose significant challenges, with targeted therapy against specific molecular targets or signaling pathways being a mainstay alongside surgical resection. Previous studies have implicated KHDRBS1 in the oncogenesis of certain human tumors such as colorectal and prostate cancers, underscoring its potential as a therapeutic target. However, the comprehensive expression pattern of KHDRBS1 in hepatocellular carcinoma (HCC) warrants further exploration.

**Methods:**

Integrating and analyzing multi-omics, multi-cohort data from public databases, coupled with clinical samples and molecular biology validation, we elucidate the oncogenic role of KHDRBS1 in HCC progression. Additionally, leveraging HCC single-cell sequencing data, we segregate malignant cells into KHDRBS1-positive and negative subsets, uncovering significant differences in their expression profiles and functional roles.

**Results:**

Our study identifies KHDRBS1 as a tumor-promoting factor in HCC, with its positivity correlating with tumor progression. Furthermore, we highlight the clinical significance of KHDRBS1-positive malignant cells, aiming to further propel its clinical utility.

**Conclusion:**

KHDRBS1 plays a key role in HCC development. This study provides crucial insights for further investigation into KHDRBS1 as a therapeutic target in HCC.

## 1 Introduction

Hepatocellular carcinoma (HCC), a highly lethal malignancy worldwide, has long been a focal point in the medical community (1). Treatment modalities for HCC have evolved from traditional surgical resection, radiotherapy, and chemotherapy to more refined approaches such as molecular targeted therapy and immunotherapy. Molecular targeted agents like sorafenib have become standard therapy for advanced HCC patients, while immunotherapy, particularly immune checkpoint inhibitors like nivolumab, has shown promising therapeutic effects in select patients (2, 3). Nonetheless, in the realm of non-surgical treatment, the quest for effective molecular biomarkers as therapeutic targets remains crucial, necessitating further research for validation.

The application of multi-omics technologies, including genomics (4), transcriptomics (5), proteomics, metabolomics, and epigenetics, has brought new dimensions to HCC research (6). Genomic deep sequencing has revealed crucial gene mutations and alterations in signaling pathways associated with HCC, such as TP53, CTNNB1, and LRP1B, which play pivotal roles in patient prognosis evaluation and therapeutic strategies (7–9). Transcriptomic analyses have unveiled bulk and single-cell RNA expression profiles in HCC, aiding in understanding tumor behavior and molecular mechanisms (10–12). Proteomic approaches allow the analysis of protein levels in HCC, including expression, modification, and interactions (13, 14). Metabolomics plays a significant role in studying tumor metabolic reprogramming, aiding in exploring metabolic pathways associated with HCC occurrence, progression, and drug resistance (15–17). Epigenetic studies have elucidated the role of epigenetic changes such as DNA methylation and histone modifications in HCC development (18).

KHDRBS1, a protein with a KH homology domain and signaling functions (19). Aberrant expression of KHDRBS1 has been observed in various cancers, garnering widespread attention in cancer research (20–23). KHDRBS1 has gained large attention by its abnormal expression in cancer, includes breast cancer and lung adenocarcinoma (24–26). Additionally, in neurobiology, the role of KHDRBS1 has been of interest, especially in neurodevelopment and neurodegenerative diseases (27, 28). Collectively, KHDRBS1 is considered a promising therapeutic target in various diseases, but its role may vary among different diseases, especially in complex solid tumors.

In this study, we conducted a multi-omics analysis of the molecular characteristics of KHDRBS1 in human cancers. Utilizing multiple HCC cohorts from various public databases along with clinical samples and experimental validation, we demonstrated the significant role of KHDRBS1 as an oncogene in HCC. Furthermore, we extended our understanding of the functional role of KHDRBS1 in malignant cell populations within HCC using single-cell data, which is crucial for targeting key cell populations in therapeutic strategies. These findings advance our understanding of the role of KHDRBS1 in human cancers and provide a solid research foundation and direction for studying KHDRBS1 in HCC.

## 2 Materials and methods

### 2.1 Dataset acquisition

We obtained expression profile data of two HCC microarrays, namely GSE14520 and GSE116174, from the GEO (https://www.ncbi.nlm.nih.gov/geo/). Additionally, high-throughput sequencing data of three other HCC cohorts were acquired from the following sources: TCGA-LIHC cohort from The Cancer Genome Atlas (TCGA) (29), LIHC-CN from a previous study, and HCCDB18, where sequencing data were downloaded from the HCCDB database. Furthermore, pan-cancer analysis data were obtained from the UCSC Xena browser (https://xenabrowser.net/). In addition, proteomic data for various cancer types were retrieved from the CPTAC database (https://proteomics.cancer.gov/programs/cptac). The mRNA expression data for cell lines were retrieved from BioGPS (http://biogps.org) and Cancer Cell Line Encyclopedia (CCLE, https://sites.broadinstitute.org/ccle/).

### 2.2 Prognosis analysis for KHDRBS1

Based on the median expression level of KHDRBS1, patients from various cancer types were stratified into groups of high KHDRBS1 expression and low KHDRBS1 expression. Subsequently, Cox proportional hazards analysis was conducted using the ‘survival’ and ‘survminer’ packages in R (30, 31), followed by the generation of Kaplan-Meier survival curves to investigate the association between KHDRBS1 expression and patient prognosis (32).

### 2.3 Single-cell analysis

We acquired eight sets of single-cell RNA sequencing data from the GSE149614 dataset, derived from four individuals diagnosed with HCC (HCC05T, HCC05N, HCC04T, HCC04N, HCC03T, HCC03N, HCC06T, HCC06N), encompassing both tumor and non-tumor tissues. The single-cell sequencing data were analyzed using the “Seurat” package (33). Initially, quality control was performed, retaining only genes with mitochondrial content below 20%. Subsequently, highly variable genes were identified for further analysis, and batch effects between eight samples were mitigated using the “Harmony” package. Cell clustering was then constructed utilizing the “FindClusters” and “FindNeighbors” functions (34). Subsequent annotation of cell clusters was carried out based on specific marker genes. Specifically, marker genes were utilized as follows: CD3D, CD8A, CD8B, GZMA for T cells; CD163, CD68, CD14 for macrophages; KLRF1, KLRD1 for NK cells; CD19, MS4A1, CD79A for B cells; MZB1, JSRP1 for plasma cells; EPCAM, KRT19 for epithelial cells; ACTA2, FAP, PDGFRB, NOTCH3 for fibroblasts; PECAM1, CDH5 for endothelial cells; and ALB, TTR, APOA2 for hepatocytes. Further discrimination of normal and malignant tumor cells within cell populations was achieved using the “copyKAT” package (35). Malignant cells were categorized based on the expression of KHDRBS1 into KHDRBS1 positive malignant cells (KHDRBS1+ malignant cells, (counts [KHDRBS1]) > 0) and KHDRBS1 negative malignant cells (KHDRBS1-malignant cells, (counts [KHDRBS1]) <= 0). KEGG and GO enrichment analyses were performed on single-cell data using the “SCP” package to explore functional disparities among different cell types. An approach proposed in a previous study was adopted to analyze metabolic differences between these two cell groups (36). Finally, differential analysis and functional enrichment analysis were conducted using the “SCP” software package, followed by visualization.

### 2.4 Establishment of a malignancy marker for KHDRBS1 positivity

Differential gene analysis was conducted between malignant cells positive for KHDRBS1 and those negative for KHDRBS1. We applied a threshold (logFC > 0.2 and p.adj < 0.05) to filter genes to identify those significantly expressed in KHDRBS1-positive malignant cells. Subsequently, models were selected based on the following criteria: 1. Minimization of the required genes during model establishment to simplify its complexity; 2. Exclusion of algorithm models with a concordance index (C-index) below 0.6 during external validation to ensure model reliability (37). This process aimed to establish a reliable malignancy marker for KHDRBS1 positivity to aid in the diagnosis and treatment of associated diseases.

### 2.5 Regents and anti-bodies

Antibody of anti-KHDRBS1 was purchased from SAB (54969, College Park, US), anti-GAPDH purchased was from Proteintech (60004, Wuhan, China), crystal violet solution was purchased from Solarbio (G1063, Beijing, China), IHC kit was purchased from Zhongshan Golden Bridge Biotechnology (PV-9001, Beijing, China). Transwell and Matrigel plates were purchased from Corning Costar (3422, 354480, 24-well format, 8μm pore size, New York, USA). Lipofectamine 3000 purchased was from Invitrogen (2369247, California, USA). DMEM, PBS, and Penicillin & Streptomycin solution were purchased from BasalMedia (L110KJ, B320KJ, S110JV, Shanghai, China), and Fetal Bovine Serum was purchased from ExCell Bio (FSP500, Shanghai, China). Cell Counting Kit-8 solution was purchased from MCE (HY-K0301, New Jersey, US).

### 2.6 Cell culture

The SK-HEP-1 cell line was procured from Servicebio (Wuhan, China). To induce overexpression of KHDRBS1 in SK-HEP-1 cells, transfection was performed using KHDRBS1 overexpression plasmids and corresponding control plasmids obtained from Miaoling Biology (Wuhan, China). Furthermore, SK-HEP-1 cell lines with KHDRBS1 knockdown were established employing the CRISPR-Cas system. Cell cultures were maintained in DMEM medium supplemented with 10% FBS and 1% antibiotics, and were housed in a humidified incubator set at 37°C with 5% CO_2_ (38, 39).

### 2.7 CRISPR

All oligos, sgRNA, and primers used in this study are listed in **Supplementary Table 1**. The pLV3-CMV-KHDRBS1-EGFP-Puro plasmid and its corresponding control, pLV3-CMV-EGFP-Puro, were procured from Miaoling Biology (Wuhan, China). KHDRBS1 sgRNA was custom-designed, synthesized, and then ligated into the lentiCRISPR-EGFP vector. This construct was employed to generate the lentiCRISPR-EGFP sgRNA construct, which was subsequently verified by DNA sequencing. Transfections were carried out using.

### 2.8 Transwell

Migration and invasion assays were conducted post-logarithmic growth phase of cells. For migration assays, 7×10^4^ cells were seeded into the upper chamber of Transwell plates, while for invasion assays, 1×10^5^ cells were added into the upper chamber of Matrigel-coated plates (9). Cells were cultured in serum-free DMEM medium in the upper chamber, whereas the lower chamber was filled with DMEM medium containing 10% serum, followed by 36 hours of incubation. Subsequently, cells were stained with crystal violet solution and images were captured using a Leica microscope. Finally, data analysis was performed using Image J software. Each experiment was repeated three times to ensure the reliability of the results.

### 2.9 Cell proliferation assay

To assess cellular proliferation activity, cells were initially seeded in a 96-well plate at a density of 3 × 10^3^ cells per well and observed at different time points (24, 48, 72, and 96 hours). Subsequently, 10μl of Cell Counting Kit-8 (CCK-8) solution and 100μl of DMEM culture medium containing 10% serum were added to each well, followed by an incubation at 37°C for 4 hours. Then, absorbance was measured at 450 nm using a microplate reader to evaluate cellular metabolic activity and viable cell count. Each experiment was performed in triplicate to ensure the reliability of the results.

### 2.10 Wound healing assay

Cells are seeded at a density of 1×10^6^ cells per well in a 6-well plate to study their migration and wound healing capabilities. Upon reaching confluency, a uniform gap is created in the cell monolayer using a cell scraper. The cells are then incubated in DMEM medium enriched with 10% serum. Images of the scratch are taken at 0 and 24 hours after the scratch is made, utilizing a microscope. The widths of the scratch at these time points are measured and analyzed using Image J software to evaluate the cells’ migration and wound healing efficiency.

### 2.11 Western blot

In the protein immunoblotting experiment, cellular samples are initially treated with RIPA lysis buffer and protease inhibitors to extract proteins. Subsequently, the protein concentration is determined, and samples are prepared accordingly. Proteins are separated by SDS-PAGE gel electrophoresis, followed by transfer onto a PVDF membrane. Following protein transfer, the PVDF membrane is blocked with 5% non-fat milk powder and then incubated overnight at 4°C with specific primary antibodies. Specific binding of primary antibodies to target proteins allows detection of protein-antibody complexes using specific secondary antibodies and chemiluminescent detection. Finally, protein imaging is performed using an imaging system, and protein expression levels are quantitatively analyzed using Image J software.

### 2.12 Quantitative PCR

In quantitative PCR experiments, total RNA is first extracted using TRIzol, followed by reverse transcription to generate cDNA. The synthesized cDNA serves as a template for PCR amplification, with SYBR Green nucleic acid probes used to monitor fluorescence intensity in each PCR cycle (40). The relative expression levels of target genes are calculated by comparing the threshold cycle (Ct) values of fluorescence signals in different samples (41).

### 2.13 Immunohistochemistry

HCC specimens were initially fixed in a solution containing 10% buffered formalin for a duration of 24 hours. Following fixation, tissue samples underwent dehydration and embedding in low-melting-point paraffin to prepare paraffin sections. Subsequently, paraffin sections were consecutively sliced into fragments with a thickness of 4-5 micrometers, affixed onto glass slides, and subjected to deparaffinization and rehydration to eliminate paraffin. Citrate repair solution was then utilized to repair tissue antigens under high temperature (100°C) and pressure (800W) conditions for 2 minutes. Following cooling, sections were treated with a peroxidase inhibitor for 10 minutes at room temperature to block endogenous peroxidase activity. Subsequently, sections were incubated overnight with specific primary antibodies at 4°C. The following day, staining of sections was performed using secondary antibodies and DAB substrate to produce chromogenic products. Finally, stained sections were dehydrated, cover slipped, and observed under a microscope for image capture. ImageJ software was employed for the quantitative analysis of optical density in the sections for IHC analysis.

### 2.14 Statistical analysis

The statistical analysis was conducted using R software version 4.1.3, while experimental data were analyzed using Graphpad and Image J (versions 9.4.0 and 1.8.0, respectively). In cellular experiments, a t-test was employed to assess differences between two groups, with statistical significance determined at p < 0.05.

## 3 Results

### 3.1 KHDRBS1 expression levels in cancer

**Figure 1:**
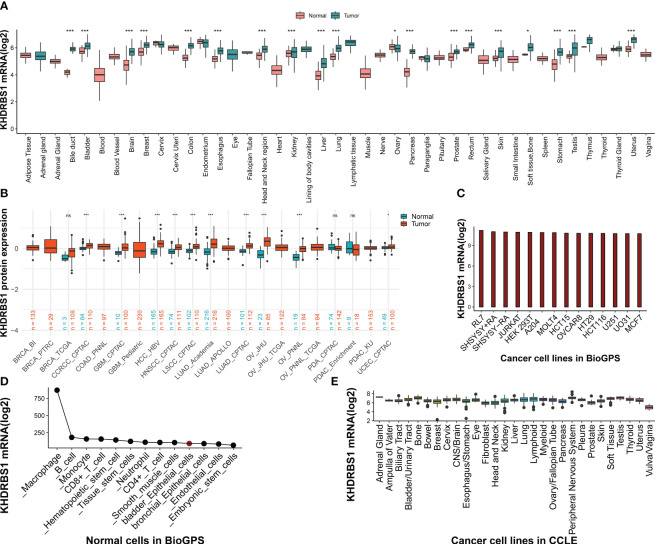
Expression Patterns of KHDRBS1 in Pan-Cancers. **(A)** Analysis of KHDRBS1 mRNA expression across a variety of cancers, using data integrated from TCGA and GTEx databases. **(B)** Examination of KHDRBS1 protein levels across 13 different cancer types, based on information from the CPTAC database. **(C)** Assessment of KHDRBS1 expression in various cancer cell lines, as recorded in the BioGPS database. **(D)** Evaluation of KHDRBS1 expression in normal cell lines, with data sourced from BioGPS. **(E)** Investigation of KHDRBS1 expression in cancer cell lines, utilizing the CCLE database. ns, *p* ≥ 0.05; *p < 0.05; ***p < 0.001.

To elucidate the fundamental expression patterns of KHDRBS1, an extensive investigation into the gene expression of KHDRBS1 was initially conducted. The differential expression of KHDRBS1 mRNA between normal and tumor tissues was examined, revealing that in the majority of cancer types, the expression levels of KHDRBS1 mRNA were significantly elevated in tumor tissues compared to normal tissues (**Figure 1A**). This observation underscores the pivotal role of KHDRBS1 in the onset and progression of tumors. Leveraging protein expression data from the CPTAC database, we investigated the differential expression of KHDRBS1 protein and identified a significant increase in KHDRBS1 protein levels across specific cancer types, including lung adenocarcinoma, ovarian cancer, and HCC, among a total of 10 cancer types examined. Conversely, in the remaining three cancer types, KHDRBS1 expression did not exhibit significant variation. This detail accentuates the heterogeneity of KHDRBS1 involvement in cancer, emphasizing its potential as a biomarker or therapeutic target within distinct oncological contexts (**Figure 1B**). Moreover, the expression of KHDRBS1 mRNA was relatively stable across various cancer cell lines, albeit with some fluctuations (**Figure 1C**). In contrast, among different cell types within normal cell lines, such as in macrophages, the highest expression levels of KHDRBS1 mRNA were recorded (**Figure 1D**). Lastly, the CCLE database revealed that the expression levels of KHDRBS1 mRNA in these cancer cell lines were also relatively stable, without significant fluctuations (**Figure 1E**).

### 3.2 Prognostic role of KHDRBS1 in HCC

**Figure 2:**
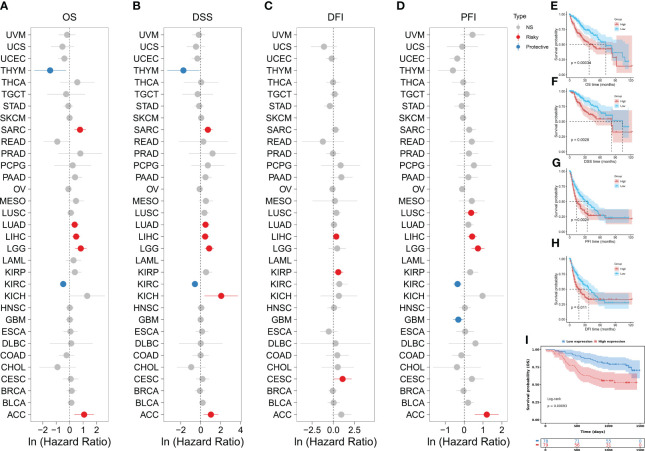
Comprehensive analysis of KHDRBS1 expression and its prognostic significance across various cancer types, with a specific focus on HCC. Forest plots illustrate the univariable Cox regression analysis of KHDRBS1 expression across 33 TCGA cancer types, covering **(A)** Overall Survival (OS), **(B)** Disease-Specific Survival (DSS), **(C)** Progression-Free Interval (PFI), and **(D)** Disease-Free Interval (DFI). Kaplan-Meier analysis evaluates the relationship between KHDRBS1 mRNA expression and **(E)** Overall Survival (OS), **(F)** Disease-Specific Survival (DSS), **(G)** Progression-Free Interval (PFI), and **(H)** Disease-Free Interval (DFI) in patients with HCC in the TCGA-LIHC cohort. Additionally, the Kaplan-Meier analysis of **(I)** Overall Survival (OS) for KHDRBS1 protein levels in HCC patients from the CPTAC database is also included.

We further investigated the association between KHDRBS1 gene expression levels and survival rates in human cancer patients. In the overall survival (OS) analysis, elevated expression of KHDRBS1, as observed in liver cancer (LIHC), was identified as an adverse prognostic factor for patients (**Figure 2A**). Consistent with OS findings, results from disease-specific survival (DSS) and disease-free interval (DFI) analyses indicated poorer disease-specific survival outcomes in liver cancer patients with high KHDRBS1 expression (**Figures 2B, C**). Subsequent progression-free interval (PFI) analysis reinforced these observations, underscoring the potential of KHDRBS1 as a viable biomarker for liver cancer (**Figure 2D**). Furthermore, detailed survival curve analysis elucidated the correlation between KHDRBS1 mRNA expression levels and various clinical outcomes in HCC patients (**Figures 2E–H**). Finally, by delving into the specific impact of KHDRBS1 protein levels on prognosis, our study revealed an association between elevated protein expression of KHDRBS1 and poorer prognosis in HCC patients (**Figure 2I**).

### 3.3 Epigenetic landscape of KHDRBS1

**Figure 3:**
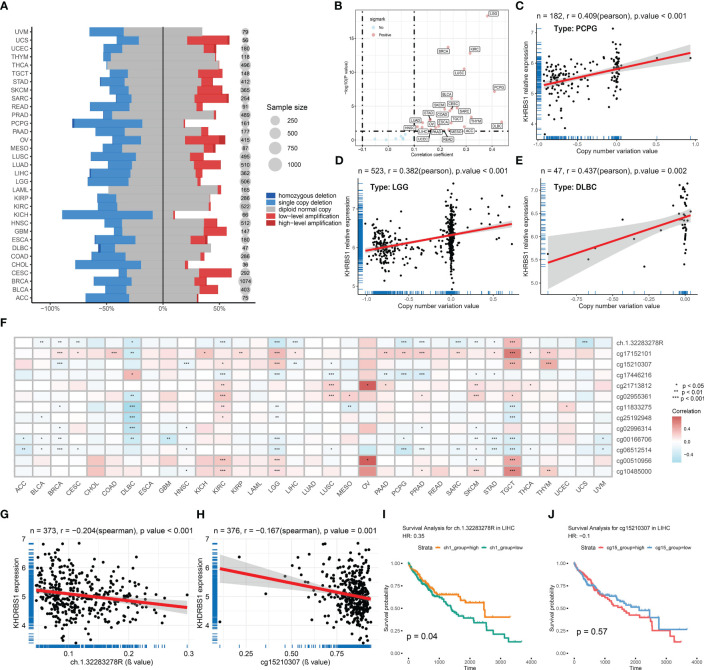
KHDRBS1 is regulated by copy number amplification and DNA methylation. **(A)** DNA copy number variation analysis of KHDRBS1 in 33 cancer types. **(B)** The mRNA expression level of KHDRBS1 is positively correlated with the CNV level in most tumors. Correlation analysis of KHDRBS1 expression and βvalue in the **(C)** PCPG, **(D)** LGG, and **(E)** DLBC datasets. **(F)** DNA methylation analysis of KHDRBS1 in 33 cancer types. The mRNA expression level of KHDRBS1 is significantly negatively correlated with the βvalue of its methylation site **(G)** ch.1.32283278R, as well as **(H)** cg15210307. **(I)** Patients with HCC exhibiting a high βvalue of the ch.1.32283278R site have better overall survival. **(J)** The βvalue level of cg15210307 is not significantly associated with the prognosis of HCC patients. *p < 0.05; **p < 0.01; ***p < 0.001.

Our investigation unveils the association between aberrant expression of KHDRBS1 gene and tumor prognosis, yet the precise mechanisms remain elusive. To elucidate the underlying mechanisms behind this dysregulated expression, we analyzed copy number variations and DNA methylation changes. At the genomic level, the distribution of KHDRBS1 gene copy number variations across different cancer types were delineated (**Figure 3A**). Notably, KICH exhibits the highest proportion of KHDRBS1 gene copy loss, whereas CESC shows the highest proportion of KHDRBS1 gene amplification. These copy number losses and amplifications occur across all tumors. Additionally, in pan-cancer analysis, a significant positive correlation between KHDRBS1 gene copy number and KHDRBS1 mRNA expression levels was observed (**Figure 3B**). Furthermore, we visually depicted the most significant correlations between KHDRBS1 gene copy number and KHDRBS1 mRNA expression levels in three tumors, namely PCPG, LGG, and DLBC (**Figures 3C–E**).

At the epigenetic level, through pan-cancer analysis, we revealed the correlation between KHDRBS1 mRNA expression levels and DNA methylation sites across different cancer types (**Figure 3F**). It was found that the methylation levels of sites ch.1.32283278R and cg15210307 exhibit a significant negative correlation with KHDRBS1 mRNA expression levels (**Figures 3G, H**). Particularly, the methylation level of site ch.1.32283278R is associated with the prognosis of HCC patients, with higher methylation levels correlating with better prognostic outcomes (**Figure 3I**). However, the methylation level of site cg15210307 shows no significant correlation with the prognosis of HCC patients (**Figure 3J**).

### 3.4 KHDRBS1 infects HCC immune evasion

**Figure 4:**
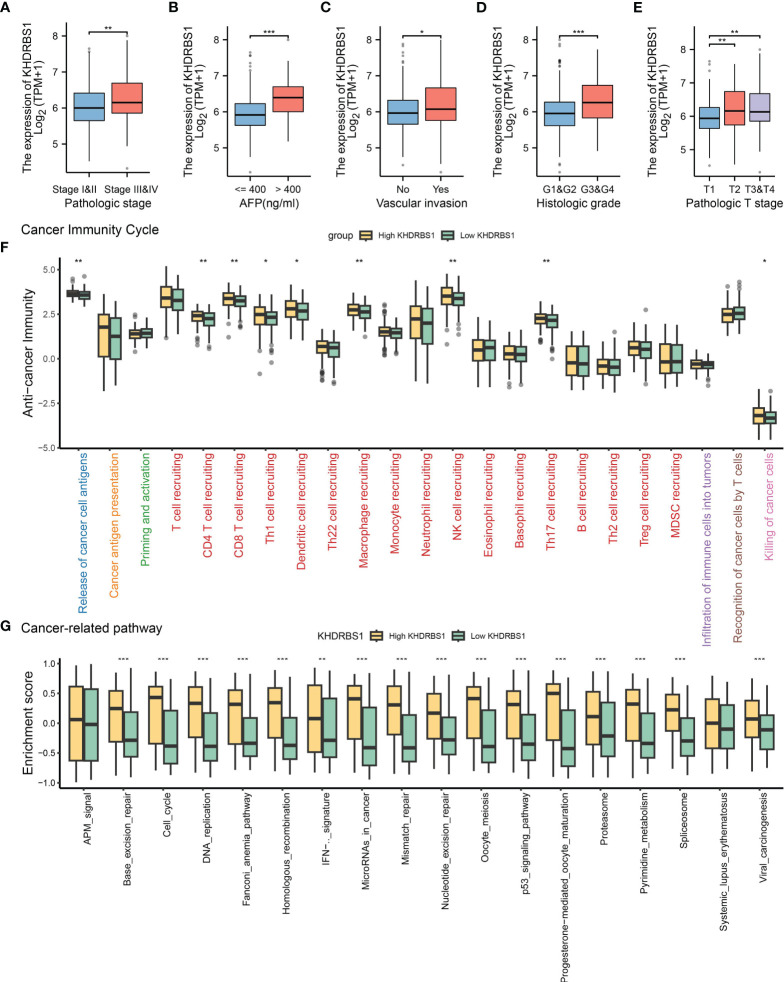
Analysis of KHDRBS1 expression and its clinical implications in liver cancer from the TCGA-LIHC cohort. **(A)** KHDRBS1 expression across different pathological stages in the TCGA-LIHC cohort. **(B)** KHDRBS1 expression levels in relation to AFP in the TCGA-LIHC cohort. **(C)** KHDRBS1 expression in cases with vascular invasion in the TCGA-LIHC cohort. **(D)** Variation of KHDRBS1 expression across different histological grades in the TCGA-LIHC cohort. **(E)** KHDRBS1 expression in various T stages of liver cancer in the TCGA-LIHC cohort. **(F)** Comparison of cancer immunity cycle steps between high and low KHDRBS1 expression groups in the TCGA-LIHC cohort. **(G)** Differential enrichment scores of cancer-related pathways between high and low KHDRBS1 expression groups in the TCGA-LIHC cohort. *p < 0.05; **p < 0.01; ***p < 0.001.

The association between KHDRBS1 gene expression and clinical pathological characteristics, tumor immune features, and tumor-related pathways in HCC patients was extensively investigated utilizing TCGA-LIHC data. Regarding pathology, tumors of higher grades exhibited significantly elevated KHDRBS1 expression compared to lower-grade tumors (**Figures 4A, E**). Furthermore, patients with serum alpha-fetoprotein (AFP) levels exceeding 400 ng/ml showed higher KHDRBS1 expression compared to those with AFP levels below 400 ng/ml (**Figure 4B**). Further analysis indicated significantly elevated KHDRBS1 expression in HCC patients with vascular invasion compared to those without vascular invasion (**Figure 4C**). Similarly, higher histological grades were associated with increased KHDRBS1 expression compared to lower grades (**Figure 4D**). These findings suggest a positive correlation between KHDRBS1 expression and HCC malignancy.

Subsequently, by exploring the relationship between KHDRBS1 expression and various stages of the tumor immune cycle, we further elucidated the role of KHDRBS1. Results revealed varied levels of immune activity across multiple stages in the high KHDRBS1 expression group, particularly in anti-tumor immune cycles, including recruitment and infiltration of T cells and NK cell infiltration (**Figure 4F**). These findings suggest that KHDRBS1 may influence HCC progression by modulating immune cell infiltration in the tumor microenvironment. Moreover, our investigation delineated the association between KHDRBS1 expression and oncogenic pathways. Notably, heightened KHDRBS1 expression significantly correlates with the activation of essential pathways implicated in cancer progression, encompassing the cell cycle, DNA replication, cellular migration, and angiogenesis (**Figure 4G**).

### 3.5 Prognostic value of KHDRBS1 in HCC: validation in independent cohorts

**Figure 5:**
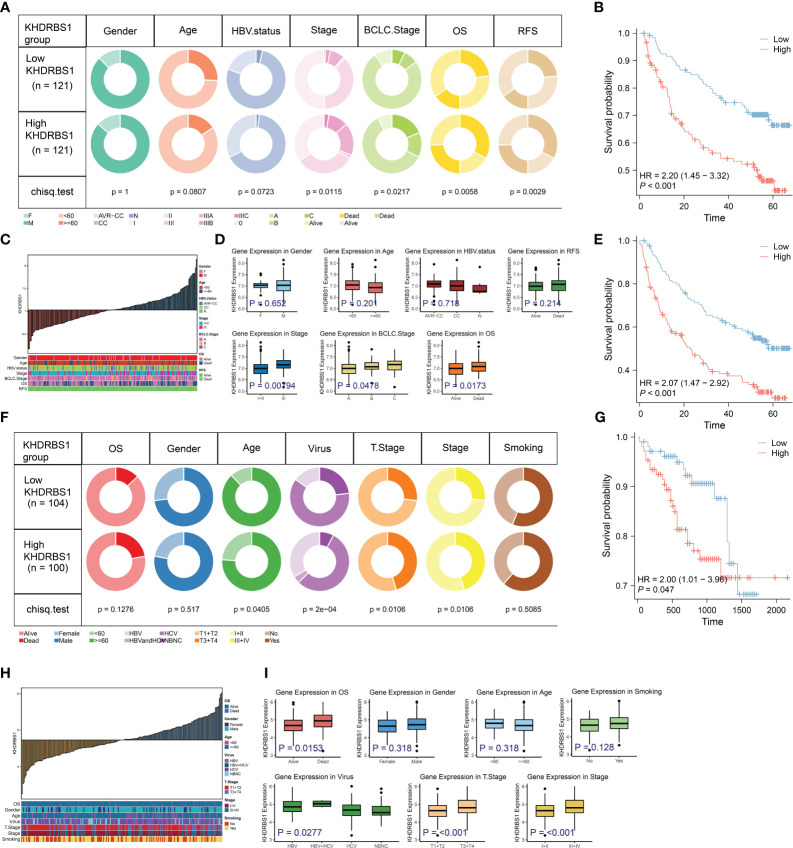
Analysis of KHDRBS1 expression and its clinical implications in liver cancer from the validation cohort. **(A)** Analysis of clinical characteristics in patients with high *vs*. low KHDRBS1 expression in the GSE14520 cohort. **(B)** Patients with HCC exhibiting high KHDRBS1 expression have a worse OS in the GSE14520 cohort. **(C)** clinical characteristics distribution across varying levels of KHDRBS1 expression in the GSE14520 cohort. **(D)** Higher levels of KHDRBS1 expression observed in advanced pathological and BCLC stages. **(E)** Patients with HCC exhibiting high KHDRBS1 expression have a worse RFS in the GSE14520 cohort. **(F)** Analysis of clinical characteristics in patients with high *vs*. low KHDRBS1 expression in the HCCDB18 cohort. **(G)** Patients with HCC exhibiting high KHDRBS1 expression have a worse OS in the HCCDB18 cohort. **(H)** Clinical characteristics distribution across varying levels of KHDRBS1 expression in the HCCDB18 cohort. **(I)** Higher levels of KHDRBS1 expression observed in advanced pathological and T stages.

In order to enhance the credibility of KHDRBS1 as a potential oncogenic gene in hepatocellular carcinoma (HCC), validation analyses were performed on two separate supplementary cohorts to confirm the correlation between KHDRBS1 expression levels and tumor-associated characteristics. Initially, an analysis of KHDRBS1 data from the GSE14520 cohort was performed (**Figures 5A–E**). Correlations were observed between KHDRBS1 gene expression and patients’ OS and RFS (**Figures 5B, E**). Additionally, distribution relationships between KHDRBS1 expression levels and clinical case features were further demonstrated through stacked bar charts (**Figure 5C**). Further studies comparing KHDRBS1 gene expression levels across different clinical feature groups (including staging, HBV status, BCLC staging, and OS) showed significant differences in KHDRBS1 expression levels in certain clinical features, such as BCLC staging and OS (**Figure 5D**). Subsequently, an analysis of KHDRBS1 data from the HCCDB18 cohort was conducted (**Figures 5F–I**). Significant associations were observed between KHDRBS1 gene expression and patients’ tumor T stage and viral infection status (**Figure 5F**). Further survival curve analyses depicted higher prognostic levels among patients with low KHDRBS1 expression compared to those with high expression (**Figures 5G, H**). Finally, comparative box plots across various clinical features revealed significant differences in KHDRBS1 gene expression levels under certain clinical conditions such as viral infection status and tumor T stage (**Figure 5I**). The results of the multivariate regression analysis in both cohorts show that KHDRBS1 indeed can serve as an independent prognostic marker for HCC (**Supplementary Table 1**).

### 3.6 KHDRBS1 enhances HCC cells’ proliferation and migratory capabilities

**Figure 6:**
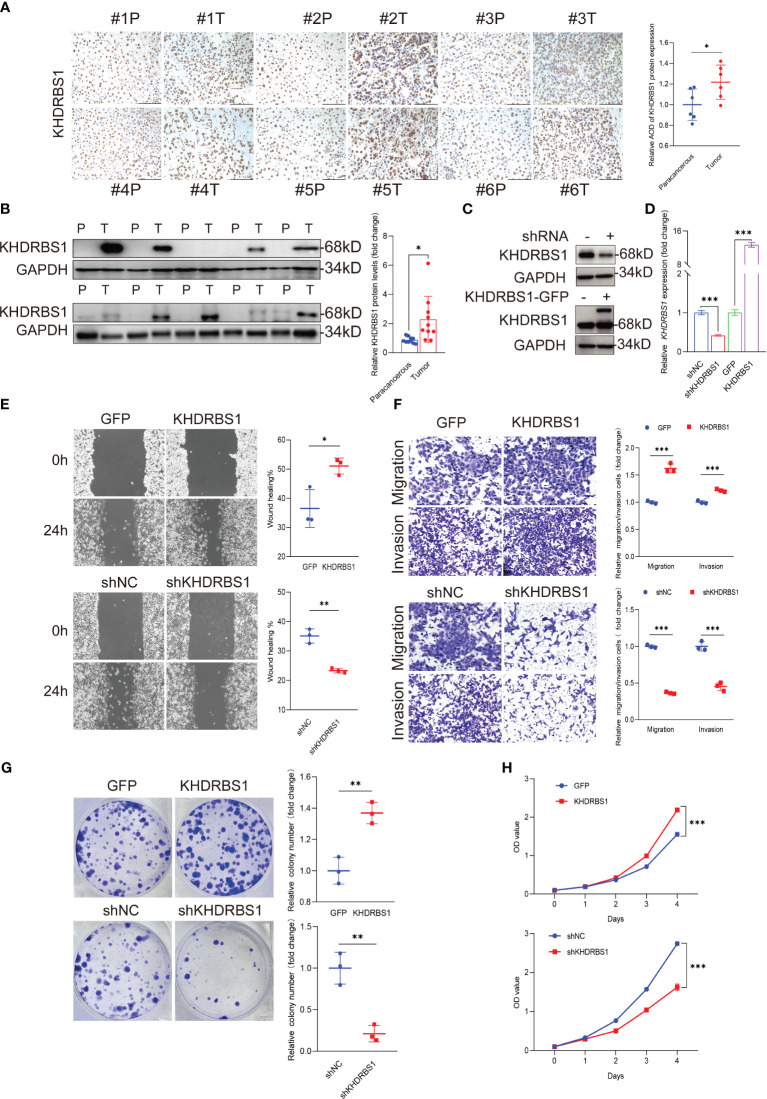
KHDRBS1 Localization and Functional Analysis in Liver Cancer. The localization **(A)** and protein expression **(B)** of KHDRBS1 were examined using immunohistochemical (IHC) and Western blot in clinical samples of liver cancer patients (n=6, T=tumor, P=para-cancerous). The efficiency of KHDRBS1 overexpression or knockdown in SK-HEP-1 cells was assessed using **(C)** Western blot and **(D)** quantitative PCR. The effects of changes in cellular KHDRBS1 expression on **(E)** wound healing, **(F)** migration and invasion, **(G)** colony formation, **(H)** cell viability were evaluated at the specified time point. Data are represented as mean ± SEM. Immunohistochemistry-stained samples visualized under light microscopy at 200× magnification. Scale bars represent 200 µm. *p < 0.05; **p < 0.01; ***p < 0.001.

To investigate the role of KHDRBS1 in HCC, we analyzed the differential expression of KHDRBS1 in clinical HCC tissues compared to adjacent non-cancerous tissues. Immunohistochemical analysis revealed significant nuclear expression of KHDRBS1, with notably elevated levels in HCC tissues compared to adjacent tissues (**Figure 6A**). These findings indicate a marked increase in KHDRBS1 expression in HCC tissues. Consistent results at the protein expression level (**Figure 6B**) further support our previous analyses from The Cancer Genome Atlas (TCGA) data. Additionally, in concordance with malignant features associated with KHDRBS1 overexpression, our study found that KHDRBS1 overexpression enhanced proliferation, clonogenicity, wound healing, migration, and invasion capabilities of SK-HEP-1 cells, while KHDRBS1 knockdown suppressed the malignant phenotype of SK-HEP-1 cells (**Figures 6C–H**). Thus, our findings strongly suggest a role for KHDRBS1 in promoting malignant characteristics of hepatocellular carcinoma.

### 3.7 Single-cell resolution reveals expression patterns of KHDRBS1 in HCC

**Figure 7:**
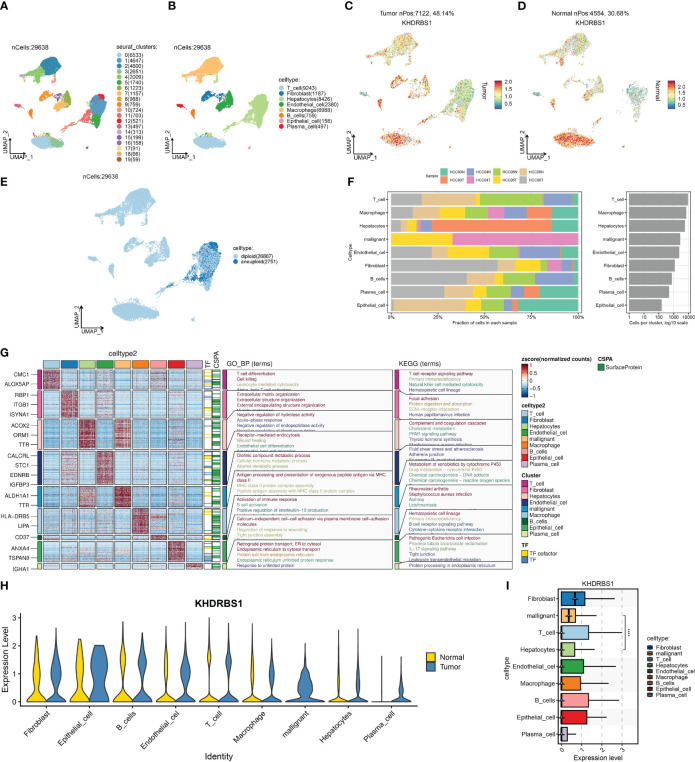
Single-cell landscape of hepatocellular carcinoma and the expression characteristics of KHDRBS1. **(A)** Distribution of 20 cell clusters was obtained and shown on a U-MAP plot; **(B)** Distribution of 20 cell clusters after cell annotation shown on a U-MAP plot; Comparison of the positivity rate of KHDRBS1 in **(C)** hepatocellular carcinoma and **(D)** adjacent normal tissues; **(E)** Distribution of malignant cells in the single-cell transcriptomic landscape as calculated by CopyKAT; **(F)** Proportional expression of different cells in different patient groups; **(G)** Enrichment analysis of differentially expressed genes from different cell types based on KEGG and GO. **(H)** Expression differences of KHDRBS1 in the same cell types between tumor and normal samples; **(I)** Expression levels of KHDRBS1 across different cell types. ****p < 0.0001.

Single-cell profiling techniques offer high-resolution insights into gene expression disparities among distinct cell populations within tissues. Analyzing single-cell sequencing data from HCC, we initially identified 20 cellular subtypes within HCC tissues and presented the results of this analysis using UMAP (**Figure 7A**). Subsequently, eight cell types were identified, including T cells, macrophages, B cells, and plasma cells (**Figure 7B**). Moreover, we compared the expression of KHDRBS1 between HCC tissues and adjacent normal tissues. The results indicated a significantly higher positivity rate of KHDRBS1 in HCC tissues compared to adjacent normal tissues (**Figures 7C, D**). Utilizing the CopyKAT algorithm, 2751 malignant cells were identified in the HCC single-cell transcriptome atlas (**Figure 7E**). Analyzing the proportions of each cell type across different samples revealed notable variations in cell proportions among patient groups (**Figure 7F**). Subsequently, we visualized differentially expressed genes for each cell type and enriched these genes through GO/KEGG analysis to identify significant pathways enriched in different cell types (**Figure 7G**). Finally, we depicted the expression levels of KHDRBS1 across different cell types (**Figures 7H, I**). Importantly, the biological functions of these pathways matched their respective cell types, further validating the accuracy of our cell annotations.

### 3.8 KHDRBS1 expression levels impact metabolic pathways and sorafenib resistance in HCC cells

**Figure 8:**
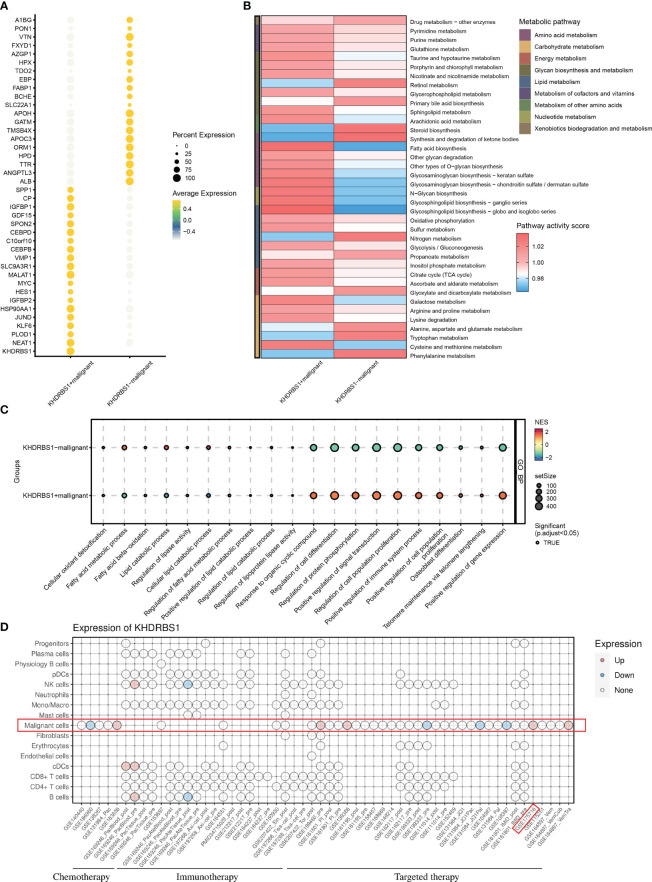
Impact of KHDRBS1 expression on malignant cells in HCC. **(A)** Differential gene expression in KHDRBS1+ *vs*. KHDRBS1- Malignant Cells. **(B)** Metabolic pathway activity differences between KHDRBS1-positive and negative malignant cells. **(C)** Biological pathway differences in KHDRBS1+ *vs*. KHDRBS1- malignant cells. **(D)** Comparative analysis of KHDRBS1 expression variations across cell types in treatment datasets.

Distinct gene expression disparities were observed between KHDRBS1-positive and KHDRBS1-negative HCC cells. **Figure 8A** illustrates the top 15 genes with the most pronounced expression changes across these two groups of HCC cells. Notably, known oncogenes such as MYC1 exhibited significant upregulation in KHDRBS1-positive HCC cells. Additionally, an in-depth analysis of metabolic pathway activities in these two sets of HCC cells was conducted. The heatmap depicted a marked increase in metabolic activity in KHDRBS1-positive HCC cells across multiple metabolic pathways, including carbohydrate and amino acid metabolism (**Figure 8B**). Additionally, analysis results from Bulk data also indicate that KHDRBS1 is associated with metabolic activity (**Supplementary Table 2**). Furthermore, comparisons of biological pathway variances between KHDRBS1-positive and KHDRBS1-negative HCC cells were made. The analyses revealed heightened metabolic activity and proliferative capacity in KHDRBS1-positive HCC cells. Moreover, active biological processes, particularly in protein phosphorylation and cell signaling pathways, were observed in KHDRBS1-positive HCC cells, underscoring the significance of signaling mechanisms in these cells. Additionally, an upregulation of immune system processes was noted in KHDRBS1-positive HCC cells, suggesting unique mechanisms for immune response modulation. Furthermore, pathways associated with cell longevity were enriched in KHDRBS1-positive HCC cells (**Figure 8C**). Lastly, drug sensitivity analysis was performed. It was observed that KHDRBS1 expression was significantly elevated in sorafenib-resistant malignant HCC cells compared to conventional malignant HCC cells (**Figure 8D**).

### 3.9 Development and validation of a KHDRBS1^+^ malignancy signature for prognostic assessment in HCC

**Figure 9:**
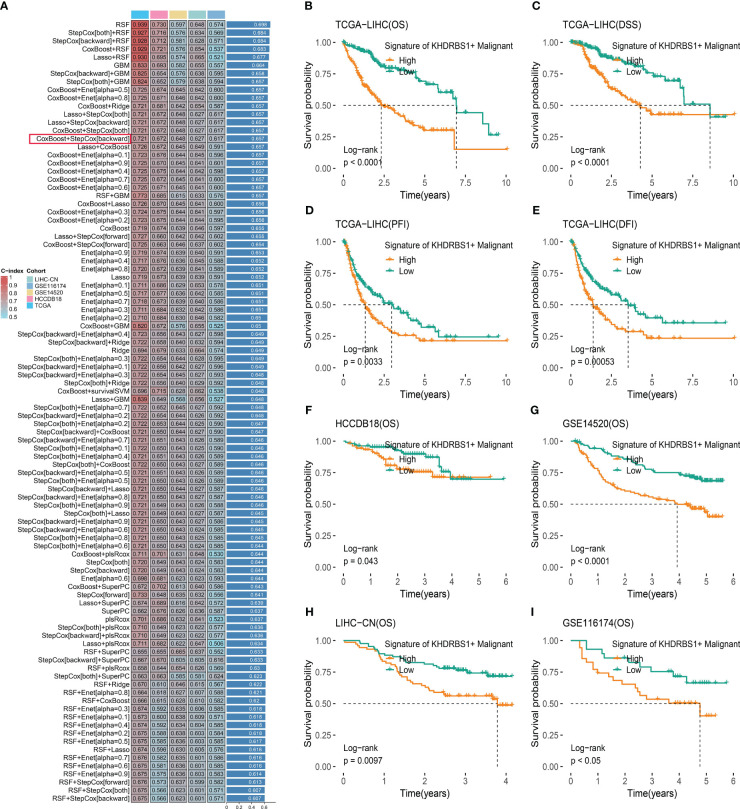
KHDRBS1+ malignant signature was developed and validated via the machine learning-based integrative procedure. **(A)** A total of 101 kinds of prediction models were used via a tenfold cross-validation framework, and the C index of each model across all datasets was further calculated. Kaplan-Meier curves for **(B)** OS, **(C)** DSS, **(D)** PFI, and **(E)** DFI based on the KHDRBS1+ malignant signature in the TCGA-LIHC cohort. Kaplan–Meier curves of OS according to the KHDRBS1+ malignant signature in the validation cohort of HCCDB18 **(F)**, GSE14520 **(G)**, LIHC-CN **(H)**, and GSE116174 **(I)**.

To investigate the impact of KHDRBS1+ HCC cells on patients with HCC, an analysis based on machine learning integration was conducted. We developed and validated a KHDRBS1+ malignancy signature aimed at assessing its role in the clinical outcomes of HCC. Utilizing the CoxBoost+StepCox [backward] algorithm, we constructed the KHDRBS1+ malignancy signature, which exhibited robust prognostic predictive ability across all training and external validation sets (**Figure 9A**). Subsequently, survival curves were employed to elucidate the influence of KHDRBS1+ malignancy in the prognosis of HCC patients in the TCGA-LIHC cohort. Analysis results demonstrated the effective prognostic prediction capability of the KHDRBS1+ malignancy signature for HCC patients’ survival (**Figures 9B–E**). Specifically, patients in the low-risk group exhibited significantly better prognosis compared to those in the high-risk group. Moreover, similar results were observed in the external validation cohort, where patients in the low-risk group showed markedly superior prognosis compared to those in the high-risk group (**Figures 9F–I**).

## 4 Discussion

In our study, multi-omics and single-cell analysis were used for a comprehensive analysis of KHDRBS1 in HCC. Survival analysis of HCC patients was performed, establishing the association between differential KHDRBS1 expression and HCC patients’ prognosis. Furthermore, an investigation into the significant association between epigenetic modifications and KHDRBS1 expression was undertaken. These findings offer novel insights and valuable revelations for a more comprehensive understanding of the molecular pathogenesis of KHDRBS1 in HCC. Additionally, our study highlights the close correlation between KHDRBS1 expression levels and immune evasion, as well as adverse prognosis in hepatocellular carcinoma. This research elucidates the pivotal role of KHDRBS1 in HCC cancer and provides scientific groundwork for potential therapeutic strategies targeting KHDRBS1.

KHDRBS1 exhibits diverse functional roles across different cell types. In neural progenitor cells, KHDRBS1 regulates 3’ end processing of Aldh1a, preventing premature transcript recognition and premature termination, thereby promoting the expression of the functional enzyme ALDH1A3, enhancing self-renewal, and glycolytic metabolism in mouse neural progenitor cells (22). Conversely, in cancer cells, KHDRBS1 acts as an oncogene, promoting breast cancer metastasis by upregulating EPHA3 gene (24), and regulating the expression of the androgen receptor splice variant AR-V7 to promote prostate cancer growth (25). Despite these findings, our pan-cancer analysis reveals an association between high KHDRBS1 expression and favorable clinical outcomes in renal clear cell carcinoma, suggesting that KHDRBS1 may not always function as an oncogene. Its functional role may depend on specific cellular and tissue contexts, necessitating further investigation to elucidate the impact of KHDRBS1 under different physiological conditions. Additionally, our focus on KHDRBS1 expression in HCC reveals that its high expression correlates with higher disease differentiation, tumor grade, and vascular invasion, indicating its significant role in tumor invasiveness and progression. Further *in vitro* experiments demonstrate that KHDRBS1 overexpression enhances proliferation, migration, and invasion capabilities of HCC cells. This oncogenic potential is further corroborated in single-cell analyses, revealing unique tumorigenic and metabolic activities in KHDRBS1-positive malignant cells, potentially associated with resistance to targeted therapies in clinical settings. Current studies also suggest that KHDRBS1 may promote cancer by influencing cell cycle regulation and enhancing tumor cell migration and invasion (21), consistent with our findings (42).

Additionally, the expression pattern and prognostic significance of KHDRBS1 in HCC patients were validated across multiple independent cohorts. Furthermore, employing single-cell sequencing technology, we delved into the cellular heterogeneity and metabolic pathway impacts of KHDRBS1 within the tumor microenvironment. Notably, we observed a significant elevation of SPP1 expression in KHDRBS1-positive HCC cells compared to negative ones. Thus, we hypothesize that the secretion of SPP1 in regulating the HCC tumor microenvironment may be a mechanism underlying the malignant functions of KHDRBS1-positive cells, as the role of SPP1 in remodeling the tumor microenvironment has been widely reported in various cancers. For instance, in hepatocellular carcinoma, colorectal cancer, and head and neck tumors, SPP1-positive macrophages can promote the formation of an immunosuppressive tumor microenvironment, thereby limiting immune cell infiltration into the tumor (43–45). Concurrently, our analysis of the anti-tumor immune response steps also supports these findings, where high expression levels of KHDRBS1 correlate with restricted immune cell infiltration. This implies that KHDRBS1-positive HCC cells may modulate the HCC tumor microenvironment by affecting the functionality of SPP1. Additionally, our analysis of anti-tumor immune steps revealed an association between elevated KHDRBS1 expression and immune cell infiltration suppression, suggesting a role for KHDRBS1 in modulating the HCC tumor microenvironment. While previous findings shed light on the influence of KHDRBS1 on glycolytic metabolism in mouse neural precursor cells, its impact on tumor cell metabolism remains unexplored (22). The results of our study underscore the pivotal role of KHDRBS1 as a facilitator in tumor cell metabolic function (46–48). Concurrently, abnormal lipid metabolic pathways were observed across cell populations with varying levels of KHDRBS1 expression, indicating a potential link between KHDRBS1 and cellular ferroptosis and sorafenib resistance (49–52). Future investigations should delve deeper into the impact of KHDRBS1 on the HCC tumor microenvironment, holding significant implications for HCC treatment.

## 5 Conclusion

Disruption in KHDRBS1 expression is influenced by genetic mutations and epigenetic mechanisms, closely associated with prognosis in HCC patients. We also highlight its potential role in HCC progression, particularly in regulating tumor cell metabolism and facilitating tumor advancement. This study furnishes scientific rationale for considering KHDRBS1 as a novel therapeutic target. Subsequent investigations can delve into tailored therapeutic approaches targeting KHDRBS1 and validate its utility as a prognostic indicator across various cancer subtypes.

## Data availability statement

The original contributions presented in the study are included in the article/**Supplementary Material**, further inquiries can be directed to the corresponding author.

## Ethics statement

The studies involving humans were approved by Ethics Committee of Xiamen University (XMYY-2022KYSB003). The studies were conducted in accordance with the local legislation and institutional requirements. The participants provided their written informed consent to participate in this study. Written informed consent was obtained from the individual(s) for the publication of any potentially identifiable images or data included in this article.

## Author contributions

RF: Data curation, Formal analysis, Methodology, Project administration, Software, Visualization, Writing – original draft. FL: Data curation, Formal analysis, Methodology, Software, Visualization, Writing – original draft. QG: Formal analysis, Methodology, Resources, Software, Writing – original draft. DL: Software, Validation, Visualization, Writing – review & editing. ST: Resources, Visualization, Writing – review & editing. DS: Conceptualization, Funding acquisition, Investigation, Methodology, Project administration, Supervision, Validation, Writing – review & editing.
