# Supplementary figures and images for: KHDRBS1 as a novel prognostic signaling biomarker influencing hepatocellular carcinoma cell proliferation, migration, immune microenvironment, and drug sensitivity
Source: Front Immunol. 2024 Apr 10;15:1393801. doi: 10.3389/fimmu.2024.1393801 (PMC11041018; doi:10.3389/fimmu.2024.1393801)

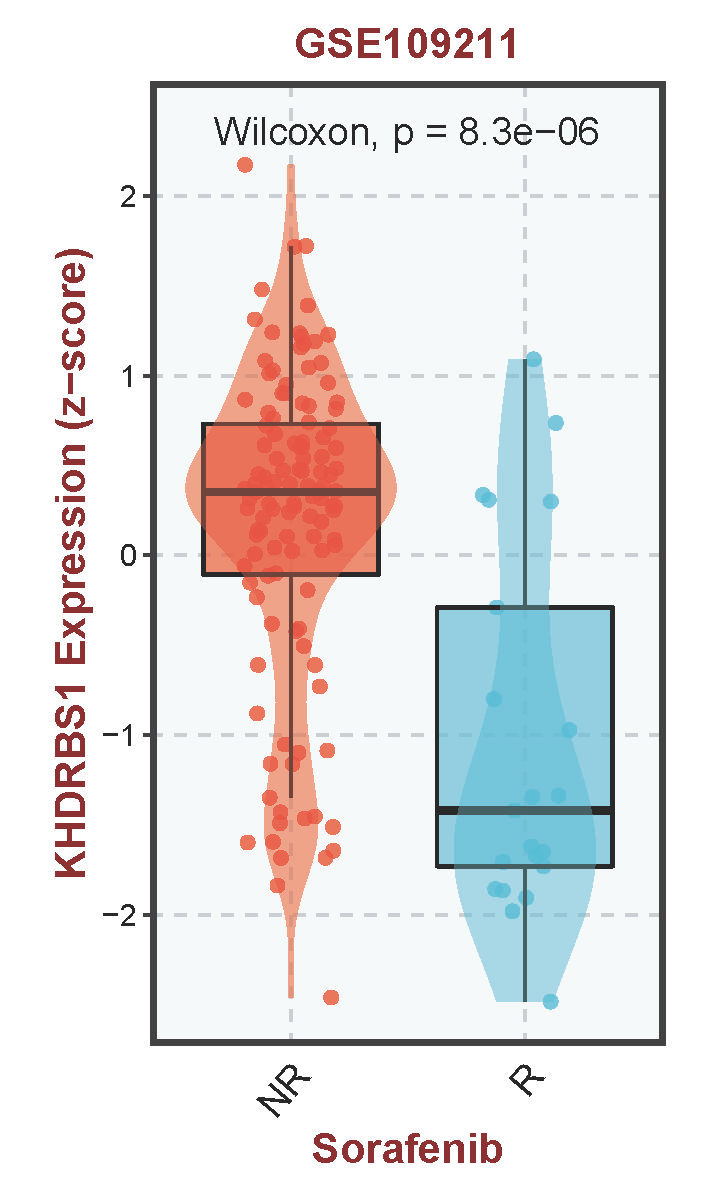

Supplement: Supplementary file 3 [file Image_1.tif]
